# Resilience, Acculturative Stress, and Family Norms Against Disclosure of Mental Health Problems Among Foreign-Born Filipino American Women

**DOI:** 10.31372/20180303.1002

**Published:** 2018

**Authors:** Andrew Thomas Reyes, Reimund Serafica, Chad L. Cross, Rose E. Constantino, Rogelio A. Arenas

**Affiliations:** a University of Nevada, Las Vegas, NV USA; b University of Pittsburgh, Pittsburgh, PA USA

**Keywords:** acculturative stress, Asian American women, disclosure of mental illness, Filipino American women, resilience

## Abstract

The present study explores the relationships between resilience, acculturative stress, and family norms against disclosure of mental health problems among foreign-born Filipino American women. The sample consisted of 159 foreign-born Filipino American women aged 18 years and above and residing in Las Vegas, Nevada, United States. Participants completed paper-and-pencil questionnaires. Results indicated high levels of resilience and moderate levels of acculturative stress. Findings also showed a significant negative correlation between resilience and acculturative stress, and a significant predictive effect of resilience on acculturative stress. We also found a significant negative relationship between resilience and family norms against disclosure of mental health problems but no significant mediating effect of resilience on the relationship between acculturative stress and family norms. This lack of significant findings related to the mediating effect of resilience on the relationship between acculturative stress and family norms against disclosure of mental illness may be due to the absence of theoretical models and research regarding the role of resilience in the context of acculturation among Filipino American women. Our findings imply the need to further explore underlying mechanisms that explain the relationships between resilience, acculturative stress, and family norms. The findings of the study also confirm the need to develop interventions and resources that ameliorate acculturative stress and promote an increase of the disclosure and reporting of mental health problems among Filipino American women.

Filipinos comprise over 19% of the Asian American population, making up the third largest Asian group in the United States after Chinese and Asian Indians (Hoeffel et al., 2012; Lopez et al., 2017). Additionally, about 69% of Filipino adults living in the United States are foreign-born (Pew Research Center, 2013), and about 60% of Filipino immigrants are women (U.S. Census Bureau, 2011).

The growing number of foreign-born Filipino American women provides an imperative to explore their experiences of migration, coping, and adjustment, and to examine mental health concerns related to U.S. Western acculturation. Many Filipino American women struggle with mental health challenges. For example, Filipino American women had the highest prevalence of moderate to severe postpartum depression (Huang et al., 2007) and the highest rate of substance abuse (Appel et al., 2011) among women of Asian American ethnicities. In addition, Filipino women reported more severe symptoms of depression and higher rates of disclosure of suicide attempts than their Filipino male counterparts (David, 2008; Espiritu and Wolf, 2001; Lim, 2011; Ying and Han, 2006). Indeed, the stress associated with acculturation to U.S. Western society has shown to be significantly related to depression and other negative mental health outcomes among Asian immigrants (Hwang and Ting, 2008; Singh et al., 2017).

In order to better understand the mental health of Filipino American women in the context of their assimilation to U.S. culture, it is necessary to examine the relationships between resilience, acculturative stress, and the influence of family norms against the disclosure of mental health problems. Resilience is a set of individual qualities that allow one to thrive despite significant challenges, and to draw on resources in order to produce desirable outcomes (Connor and Davison, 2003; Yee et al., 2007). Acculturative stress refers to behavioral responses to acculturation that indicate poor mental health, psychosomatic symptoms, and identity confusion (Berry et al., 1987). The influence of family norms is also important because many Asian Americans are reluctant to disclose mental health problems in order to prevent shame in the family (Sanchez and Gaw, 2007), resulting in low rates of help-seeking behaviors such as pursuing mental health services (Augsberger et al., 2015; Han and Pong, 2015).

## Resilience and Acculturative Stress

Within the metatheory of resilience, Richardson (2002) posits that resilience is a protective phenomenon that allows individuals to buffer the negative impact of stress and consequently develop coping processes and abilities to reintegrate disruptions during challenging times. Castro and Murray (2010) concur that resilience within the context of acculturative stress enables individuals to develop cultural adjustment from chronic stressors experienced by immigrants within a new environment. Studies confirm these claims showing a significant negative relationship between acculturative stress and resilience (Archuleta, 2015; Cheung and Yue, 2012; Miletic, 2014; Yoo et al., 2013). Studies also show the predictive effects of resilience and acculturation on depressive symptoms and negative emotions (Miller and Chandler, 2002; Yu et al., 2014). Additionally, the health risks posed by acculturative stress among Filipinos (Serafica, 2011), the buffering characteristic of resilience against stress, and the nature of resilience as a learnable phenomenon (Dyer and McGuinness, 1996; Luthar et al., 2000) provide the theoretical bases to test the relationship between resilience and acculturative stress. Despite these growing studies, the relationship of resilience and acculturative stress among Asian Americans remains somewhat unclear and needs further investigation, particularly among fast-growing groups such as Filipino American women.

## Acculturative Stress and Family Norms Against Disclosure of Mental Health Issues

This study examines the effect of acculturative stress on family norms against disclosure of mental health problems (FNAD) because studies have shown that as Asians are positively acculturated into Eurocentric U.S. culture, they develop more positive views of seeking professional mental health services (Kim and Omizo, 2003; Leong et al., 2011; Liao et al., 2005; Zhang and Dixon, 2003). On the other hand, when Asians experience acculturative stress and strongly adhere to their Asian cultural values, there tends to be an increased perceived stigma toward mental health treatment (Kim, 2007; Kim and Omizo, 2003; Miville and Constantine, 2007; Shea and Yeh, 2008). The theoretical basis of these correlations is Berry’s (1997) model of acculturation, in which stress responses occur when an individual is faced with pressures to assimilate into the dominant culture. The individual’s resources are not sufficient enough to adapt and adjust to the new cultural environment (Roysircar and Maestas, 2002). Maintaining the balance between the individual’s original culture and the dominant culture into which one is assimilating becomes challenging and results in a stress response called acculturative stress (Berry, 2006; Torres, 2010). Consequently, protective factors are utilized in order to cope with the perceived imbalance of cultural demands and available resources (Smart and Smart, 1995). For example, most Asian cultures value the belief of “saving face,” which is about preventing shame and embarrassment and preserving social integrity (Zane and Yeh, 2002). Hence, when Asian families face challenges (e.g., domestic violence, posttraumatic stress experiences, and mental health issues) that threaten their social integrity, they consequently resort to “saving face,” which usually results in the concealment of significant mental health information within families (Sue et al., 1994). Perceptions of loss of face are also related to more negative attitudes toward professional mental health assistance (Leong et al., 2011).

Therefore, it is important to explore Asian Americans’ patterns of disclosing mental health problems outside their families in order to better understand their help-seeking behaviors, particularly their approach toward mental health services (Zhang and Dixon, 2003). Liu (2009) found that stigmas surrounding mental illness and seeking mental health services prevent Asians from seeking mental health treatment. Studies have shown that acculturative stress is a significant predictor of perceived stigma for seeking help (Liao et al., 2005; Maneze et al., 2016; Prabhakar-Gippert, 2016). Conversely, acculturation into the U.S. culture has shown to be significantly related to positive attitudes toward seeking mental health services (Kung, 2003; Lor et al., 2017).

Findings about acculturation and self-disclosure of mental health concerns are inconsistent. Some studies have shown no significant relation between the two variables (Bang, 1998; Greenidge, 2007; Mo et al., 2006), while other studies involving Asian immigrants have shown that higher levels of acculturation are related to greater willingness to disclose trauma and mental health concerns (Fung and Wong, 2007; Thikeo et al., 2015). Whealin et al. (2013, 2015) found that stronger FNAD among Asian Americans were correlated with more reported posttraumatic stress disorder (PTSD) symptoms. On the other hand, other studies (Abe-Kim et al., 2007; Abdullah and Brown, 2011; Guo et al., 2015) have demonstrated that stronger family norms against disclosing mental problems are correlated with lower psychological distress and mental health problems among Asian Americans. Hence, these inconsistent findings warrant the need to further clarify the relationship between the disclosure of mental health problems and acculturative stress among Asian Americans, particularly Filipino American women.

In this study, we aimed to examine (1) levels of resilience and acculturative stress, (2) the relationships between resilience, acculturative stress, and FNAD, and (3) the potential mediating effect of resilience on the relationship between acculturative stress and FNAD among foreign-born Filipino American women.

## Methods

### Participants

We used convenience sampling to recruit a community-based sample of foreign-born Filipino American women. Eligibility criteria for the study included: 18 years of age or older, female, born in the Philippines, and living in the Las Vegas, Nevada, U.S. area. We defined first-generation participants as those who were born in the Philippines and immigrated to the United States at age 8 or older, and 1.5 generation as those who were born in the Philippines and immigrated at age younger than 8. The study had 159 participants. Most of the participants were between the ages of 35 and 64 (60.4%), employed full or part time (77.5%), first generation (91.2%), reported a personal income less than $75,000 (72.1%), and had at least a bachelor’s degree (69.2%) (Table 1).

**Table 1:** Demographic Characteristics (N and %) and Scale Scores (Mean (SD))

Value	N (%)*	CD-RISC-10	SAFE
*Age*			
18–24	11 (6.9)	29. 9 (5.28)	26.9 (9.36)
25–34	20 (12.6)	31.4 (5.55)	34.9 (17.82)
35–54	48 (30.2)	29.5 (7.16)	27.9 (17.90)
55–64	48 (30.2)	30.7 (8.59)	29.5 (18.10)
65–74	21 (13.2)	30.8 (7.49)	27.3 (15.25)
75+	11 (6.9)	32.8 (4.59)	35.0 (32.83)
[0,1–4] *Employment Status*			
Employed full time	88 (58.3)	30.25 (7.94)	29.83 (16.38)
Employed part time	29 (19.2)	30.61 (6.09)	25.04 (20.78)
Unemployed/looking for work	3 (2.0)	28.67 (7.57)	35.00 (2.00)
Student	2 (1.3)	27.00 (4.24)	24.50 (10.61)
Homemaker	5 (3.3)	29.00 (7.68)	19.20 (9.28)
Retired	22 (14.6)	32.73 (6.32)	33.14 (23.49)
Unable to work	2 (1.3)	28.50 (3.54)	26.00 (8.49)
[0,1–4] *Generational Status*			
First generation	145 (91.2)	30.71 (7.02)	29.91 (18.71)
1.5 generation	14 (8.8)	28.36 (8.93)	25.93 (14.28)
[0,1–4] *Income*			
<$10,000	15 (10.2)	31.62 (8.25)	33.57 (24.43)
$10,000–$24,999	29 (19.7)	30.00 (6.02)	30.00 (21.39)
$25,000–$49,999	40 (27.2)	30.83 (6.89)	28.00 (17.08)
$50,000–$74,999	22 (15.0)	30.86 (9.26)	32.55 (20.46)
$75,000–$99,999	18 (12.2)	31.67 (6.26)	26.33 (11.19)
$100,000–$149,999	18 (12.2)	32.47 (7.26)	27.24 (14.75)
$150,000+	5 (3.4)	34.60 (3.29)	22.80 (12.05)
[0,1–4] *Education*			
No schooling completed	1 (0.6)	15**	26**
12th grade or less	4 (2.6)	30.25 (3.78)	57.70 (23.56)
HS or equivalent	16 (10.3)	27.25 (7.49)	38.31 (25.26)
Some college, no degree	17 (10.9)	30.53 (7.79)	28.88 (15.67)
Associate degree	10 (6.4)	29.20 (7.84)	30.80 (11.70)
Bachelor’s degree	84 (53.8)	31.38 (6.71)	27.49 (17.63)
Master’s degree	22 (14.1)	31.36 (5.09)	27.14 (13.44)
Doctoral degree	2 (1.3)	34.50 (0.71)	4.00 (5.66)
*Overall*	159 (100)	30.4 (7.28)	29.6 (18.33)
*Cronbach’s α*		0.922	0.932

* Valid percent for those responding to the question.

** No SD reported as only 1 participant had no schooling completed

### Measures

#### Resilience

The Connor-Davidson Resilience Scale 10-item (CD-RISC-10, Campbell-Sills and Stein, 2007) was used to measure psychological resilience. The CD-RISC-10 comprises 10 items, and each item on the scale is rated on a 5-point Likert scale from 0 (not true) to 4 (true nearly all the time). The total score ranges from 0 to 40, with higher total scores indicating greater resilience. The scale demonstrated good construct validity and internal consistency (*α* = 0.85) during the development of the scale (Campbell-Sills and Stein, 2007). Women’s studies using the CD-RISC-10 also had good Cronbach’s alpha level of 0.86 to 0.88 (Hébert et al., 2018; Scali et al., 2012).

#### Acculturative stress

The Social, Attitudinal, Familial, and Environmental Acculturative Stress Scale (SAFE, Mena et al., 1987) was used to assess the negative stressors experienced by the foreign-born Filipino American women as they acculturated to the host culture. The 24 items in the SAFE were rated on a 6-point Likert scale from 0 (have not experienced) to 5 (extremely stressful). Total score is summed from all items with a possible score ranging from 0 to 120. Joiner and Walker (2002) established convergent and discriminant validity of SAFE through significant correlations of acculturative stress to depressive and anxiety symptoms, general life stress, and social support. Reliability of the total scale has been shown to be reasonable in different studies at Cronbach’s alpha of 0.89 (Mena et al., 1987; Joiner and Walker, 2002).

#### Family norms against disclosure of mental health problems

The variable of family norms against disclosure (FNAD) of mental health problems was developed as a result of an extensive literature review and focus groups of ethnoracially diverse military veterans in Hawaii exploring their cultural beliefs about mental health and barriers to care (Whealin et al., 2008). This measure is comprised of a single-item question: “People in my family do not talk about mental health problems with people outside of my family” (Whealin et al., 2013, 2015). Respondents rate their agreement on a 5-point scale ranging from 1 (strongly disagree) to 5 (strongly agree). The content validity of this item was evaluated by comparing responses with qualitative data of 40 veterans from different ethnoracial groups (Whealin et al., 2013).

### Procedures and Data Analyses

After obtaining ethics approval from the University of Nevada, Las Vegas to conduct the study, we started recruitment of participants in public locations, such as community centers, parks, churches, and shopping centers. Participants were provided a brief information sheet about the study, and an informed consent was obtained prior to completing the paper-and-pencil questionnaires. Participants were also provided with a quiet, comfortable space to complete the surveys. All collected data were anonymized.

Sum scores on the CD-RISC-10 and SAFE instruments were calculated prior to analysis to allow for statistical summarization and assessment. Additionally, data for the one-question item on family disclosure was categorized ordinally by response across groups of interest. Data were summarized according to each of the categorical variables of age, employment, generation, personal income, and education. General frequencies and score statistics were calculated for each of these demographic categories. Further, total score statistics and overall Cronbach’s alpha reliability measures were calculated for the CD-RISC-10 and SAFE instruments.

Inferential statistical models were used to assess potential correlations among variables, to test for significant differences in scores for the scale instruments, and to construct a mediation model. Data were tested for distributional assumptions and were found generally to meet the normality assumptions of the tests reported herein. However, we chose to report the more conservative Spearman-rank correlations in the results, although the statistical conclusions were the same using either Spearman- or Pearson-based inference. We used analysis of variance (ANOVA) to test for demographic differences in scores for each instrument. Owing to sparsity of several cells, we dichotomized demographic variables to facilitate examination by ANOVA. The dichotomous outcomes were: age (<55 years, 55+ years); employment (employed, not employed); personal income (<$50,000, $50,000+); and education (less than college degree, college degree(s)).

To assess the potential mediating effect of resilience on the relationship between acculturative stress and family norms against disclosure, we utilized mediation models, with indirect effects analyzed by way of bootstrap confidence intervals with 10,000 resamples (Hayes, 2018). All statistical analyses were conducted in SPSS (v. 25; IBM SPSS, Armonk, NY), and the mediation models were developed using the PROCESS (v. 3) macro of Hayes (2012).

## Results

### Scale Scores

The overall mean score of the CD-RISC-10 instrument was 30.4 (SD = 7.28) and had a high reliability (*α* = .922). Scores on this instrument ranged from a low of 1 to a high of 40. The overall mean score of the SAFE instrument was 29.6 (SD = 18.33) and had a high reliability (*α* = 0.932). Scores on this instrument ranged from a low of 0 to a high of 97. Correlations among scales were inconsistent. The CD-RISC-10 was significantly correlated negatively with SAFE (*r*_s_ = −0.285, *p* < 0.001) and FNAD (*r*_s_ = −0.160, *p* = 0.052). SAFE was not correlated with FNAD (*r*_s_ = 0.03, *p* = 0.765). We also calculated the frequency of responses of the FNAD (Table 2).

**Table 2:** Frequency (Row %)* Responses to the Family Disclosure Question: “People in my Family Do not Talk About Mental Health Problems with People Outside My Family”

Value	Strongly Disagree	Disagree	Neither Agree or Disagree	Agree	Strongly Agree	Total
*Age*						
18–24	0 (0.0)	3 (27.3)	3 (27.3)	4 (36.4)	1 (9.1)	11
25–34	2 (10.0)	3 (15.0)	8 (40.0)	4 (20.0)	3 (15.0)	20
35–54	8 (17.8)	10 (22.2)	12 (26.7)	10 (22.2)	5 (11.1)	45
55–64	7 (16.3)	9 (20.9)	11 (25.6)	11 (25.6)	5 (11.6)	43
65–74	5 (23.8)	3 (14.3)	6 (28.6)	4 (19.0)	3 (14.3)	21
75+	2 (22.2)	0 (0.0)	5 (55.6)	2 (22.2)	0 (0.0)	9
Total	24 (16.1)	28 (18.8)	45 (30.2)	35 (23.5)	17 (11.4)	149
*Employment Status*						
Employed full time	9 (11.1)	19 (23.5)	22 (27.2)	22 (27.2)	9 (11.1)	81
Employed part time	6 (22.2)	2 (7.4)	11 (40.7)	4 (14.8)	4 (14.8)	27
Unemployed/looking for work	0 (0.0)	0 (0.0)	2 (66.7)	1 (33.3)	0 (0.0)	3
Student	0 (0.0)	2 (100.0)	0 (0.0)	0 (0.0)	0 (0.0)	2
Homemaker	1 (20.0)	1 (20.0)	1 (20.0)	1 (20.0)	1 (20.0)	5
Retired	6 (28.6)	1 (4.8)	8 (38.1)	3 (14.3)	3 (14.3)	21
Unable to work	1 (50.0)	0 (0.0)	0 (0.0)	1 (50.0)	0 (0.0)	2
Total	23 (16.3)	25 (17.7)	44 (31.2)	32 (22.7)	17 (12.1)	141
*Generational Status*						
First generation	23 (17.0)	25 (18.5)	41 (30.4)	34 (25.2)	12 (8.9)	135
1.5 generation	1 (7.1)	3 (21.4)	4 (28.6)	1 (7.1)	5 (35.7)	14
Total	24 (16.1)	28 (18.8)	45 (30.2)	35 (23.5)	17 (11.4)	149
*Income*						
< $10,000	2 (15.4)	2 (15.4)	6 (46.2)	2 (15.4)	1 (7.7)	13
$10,000–$24,999	7 (25.0)	2 (7.1)	10 (35.7)	5 (17.9)	4 (14.3)	28
$25,000–$49,999	5 (13.2)	6 (15.8)	13 (34.2)	10 (26.3)	4 (10.5)	38
$50,000–$74,999	3 (15.0)	4 (20.0)	4 (20.0)	4 (20.0)	5 (25.0)	20
$75,000–$99,999	1 (5.6)	5 (27.8)	7 (38.9)	5 (27.8)	0 (0.0)	18
$100,000–$149,999	3 (18.8)	5 (31.3)	2 (12.5)	3 (18.8)	3 (18.8)	16
$150,000+	0 (0.0)	0 (0.0)	1 (20.0)	4 (80.0)	0 (0.0)	5
Total	21 (15.2)	24 (17.4)	43 (31.2)	33 (23.9)	17 (12.3)	138
*Education*						
No schooling completed	1 (100.0)	0 (0.0)	0 (0.0)	0 (0.0)	0 (0.0)	1
12th grade or less	0 (0.0)	0 (0.0)	2 (50.0)	1 (25.0)	1 (25.0)	4
HS or equivalent	4 (26.7)	1 (6.7)	6 (40.0)	4 (26.7)	0 (0.0)	15
Some college, no degree	4 (23.5)	5 (29.4)	6 (35.3)	2 (11.8)	0 (0.0)	17
Associate degree	0 (0.0)	4 (40.0)	3 (30.0)	3 (30.0)	0 (0.0)	10
Bachelor’s degree	12 (15.6)	12 (15.6)	23 (29.9)	17 (22.1)	13 (16.9)	77
Master’s degree	2 (10.0)	5 (25.0)	3 (15.0)	7 (35.0)	3 (15.0)	20
Doctoral degree	0 (0.0)	0 (0.0)	2 (100.0)	0 (0.0)	0 (0.0)	2
Total	23 (15.8)	27 (18.5)	45 (30.8)	34 (23.3)	17 (11.6)	146

* Valid percent for those responding to the question.

### Mediation Model

We first tested potential demographic covariates to determine if age, employment, generational status, personal income, or education were significant factors in scale scores for the SAFE, CD-RISC-10, or FNAD. Exploratory ANOVAs were used to examine differences in instrument scores. Potential predictors were all entered into saturated models. Initial models suggested no significant interaction effects, and hence main effect models were analyzed. For the CD-RISC-10 instrument, no predictors were significant (all *p* > 0.05). For the SAFE instrument, education level was significant, but with a small effect, such that those with a college degree had lower scores (*F* = 7.984, *p* = 0.005, *η*^2^ = 0.057). For the FNAD instrument, a nonparametric equivalent ANOVA on ranks also suggested a significant, but small effect, the difference owing to education (*F* = 3.97, *p* = 0.048, *η*^2^ = 0.01), with those with higher education more likely to endorse agreement that mental health problems are not discussed outside the family. However, inasmuch as these differences are inconsistent among instruments and have low effect sizes, it was not deemed appropriate or useful to consider these variables as covariates in the mediation models.

**Figure 1.**
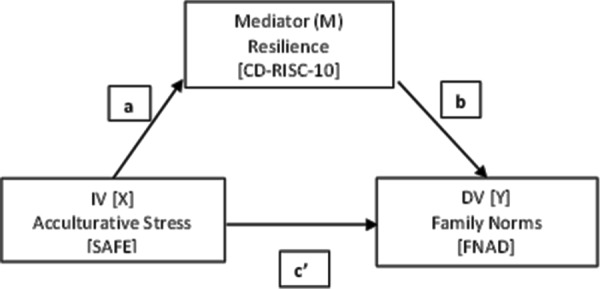
Mediation path model relating an independent variable (X) to a dependent variable (Y) through a mediator (M). Here, *a* represents the direct effect of X on M; *b* represents the direct effect of M on Y; and *c*’ represent the direct effect of X on Y (Hayes, 2018).

We explored resilience as a potential mediator between acculturative stress and family norms against disclosure of mental health issues using a mediation model (Figure 1) (Hayes, 2018). The results mirrored the correlations reported above. Scores on the SAFE instrument were significant predictors of scores on the CD-RISC-10 (*β* = −0.08, *p* = 0.018), scores on the CD-RISC-10 were not significant predictors of FNAD (*β* = −0.03, *p* = 0.061), and scores on the SAFE instrument were not significant predictors of FNAD (*β* = −0.001, *p* = 0.857). Using 95% bootstrap confidence intervals, where significance can be ascertained by noting if the interval does not contain 0, we did not find an indirect relationship of SAFE to FNAD accounting for the mediator of CD-RISC-10 (95% CI: [−.0001, 0.0055]).

## Discussion

In this study, we examined the levels of resilience and acculturative stress among foreign-born Filipino American women. We also explored the relationships between resilience, acculturative stress, and FNAD. Finally, we analyzed the potential mediating effect of resilience on the relationship between acculturative stress and FNAD.

### Level of Resilience

The resilience level of the participants, based on the overall mean score on the CD-RISC-10, indicates high resilience among foreign-born Filipino American women, and higher than among female participants of different ethnicities in other studies that used the CD-RISC-10 (Hébert et al., 2018; Scali et al., 2012; Vitale, 2015). The participants’ high level of resilience is consistent with other studies in which Asian American women had a higher level of resilience than their male counterparts (Dial, 2007; Hamid, 2007). On the other hand, there are studies showing that women of different ethnicities (i.e., Hispanic and African American women) are more resilient than Asian American women (Clauss-Ehlers et al., 2006; Kwong et al., 2015). This variability of the levels of resilience among women from different ethnicities suggests that the buffering nature of Asian American women’s resilience against stress varies across different cultural contexts (Reyes and Constantino, 2016). Hence, we explore further the effect of resilience on acculturative stress below.

### Level of Acculturative Stress

Based on the overall mean score using the SAFE, participants in this study reported a moderate level of acculturative stress, which is lower than among female participants in studies that used the same SAFE instrument (Bhargava, 2007; McGinley et al., 2010; Papazyan, 2012; Ross, 2013). The educational level of the participants (i.e., over 50% obtained a college degree and about 14% had a master’s degree) may possibly explain the moderate level of acculturative stress. Our findings showed that participants with a college degree had significantly lower levels of acculturative stress, albeit with a small effect. Studies have shown that higher educational attainment is related to lower levels of acculturative stress (Abdulahad et al., 2014; Dow and Woolley, 2011). In Berry’s, (1997) model of acculturation, an individual’s education serves as a protective factor against acculturative stress. Additionally, higher educational attainment contributes to one’s social capital (resources in social networks that facilitate goal achievement), which in turn contribute to ameliorating acculturative stress (Bhattacharya, 2011; Missingham et al., 2004). Moreover, the participants’ high level of resilience could also explain their moderate level of acculturative stress. The relationship of these two variables is further discussed in subsequent sections.

### Relationship Between Resilience and Acculturative Stress

Our analyses showed a significant negative correlation between resilience and acculturative stress, and a significant predictive of resilience on acculturative stress. These results support previous studies with findings that indicate the negative relationship between resilience and acculturative stress (Archuleta, 2015; Yoo et al., 2013; Yu et al., 2014). Our findings are also consistent with the theoretical explication of resilience as a phenomenon known to buffer the negative impact of stress (Rutter, 1993). Resilience allows individuals to overcome their challenging situations, achieve their goals, and even develop mastery over other stressful situations (Dyer and McGuinness, 1996). Additionally, resilience has shown to be a protective factor among Asian immigrants against negative mental health outcomes such as depression brought about by acculturative stress (Yoo et al., 2013; Yu et al., 2014).

While our findings show that foreign-born Filipino American women’s resilience helps alleviate stress related to acculturation, these findings are preliminary. Further exploration of the mechanisms underlying the relationship between resilience and acculturative stress are needed. For example, the moderating and mediating effects of ethnic identity, bicultural identity, gender identity, support systems, colonial mentality, experiences of racial discrimination, and overall health and well-being need to be explored in future studies. The multidirectional interaction and complex nature of the abovementioned variables must be determined in order to provide a possible explanation for the buffering mechanism of resilience on acculturative stress.

### Relationship of Family Norms with Resilience and Acculturative Stress

While we found a significant negative relationship between resilience and FNAD, there was no significant predictive effect of resilience on family norms. We also did not find a predictive effect of acculturative stress on FNAD. A possible explanation of this lack of predictive effects could be related to the use of a single-item construct to account for family norms, which may reduce the power of the statistical model, or may inadequately capture variability in this construct among participants. The variable of FNAD needs to be further explored and tested in order to fully identify its critical elements. This variable was recently tested by Whealin et al. (2013, 2015). Whealin and colleagues report significant findings at the early stage of the development of this variable. They found that stronger FNAD were associated with more PTSD symptoms reported (Whealin et al., 2013, 2015). However, their study findings were somewhat different from findings in the literature about the lower prevalence of reported mental health problems related to family norms among Asian Americans (Abe-Kim et al., 2007; Guo et al., 2015). These differing results provide the imperative to conduct further research in this area. To our knowledge, there are no studies investigating the relationship between resilience, acculturative stress, and FNAD.

### Mediating Effect of Resilience

We did not find a significant mediating effect of resilience on the relationship between acculturative stress and FNAD. The lack of significant findings related to the mediating effect of resilience on the relationship between acculturative stress and family norms against disclosure of mental illness is difficult to explain, but might be due to the lack of theoretical models and research regarding the role of resilience among Filipino American women. Other possible explanations for the lack of significant findings could be related to the use of the CD-RISC-10 (which is an abbreviated version of the original CD-RISC), the lack of predictive effect of acculturative stress on family norms, the small sample size of the study, and the need to further theoretically develop the variable of FNAD. Therefore, our results should be considered preliminary. We recommend further testing of the relationships among these variables using a larger sample size and the original 25-item version of the CD-RISC (Connor and Davison, 2003) in future research. A mixed methods design, in which qualitative and quantitative data collection and data analyses are integrated, and/or qualitative data are quantified and quantitative data are qualified (Creswell et al., 2011), is needed in order to explore particular norms, beliefs, and practices that Filipino women hold against disclosing mental health problems outside their families. The outcomes of a mixed methods study could further inform us about the variable of FNAD. We also recommend using qualitative research methods to further explore the processes that explain the relationship between the different variables. Through qualitative methods, we also recommend examining strategies that foreign-born Filipino American women use to address their mental health problems.

### Limitations

Our findings should be interpreted in light of the study limitations. The sample of the study was limited to foreign-born Filipino women in the Las Vegas, Nevada area. Expanding the sample to other geographical areas in the United States where there are high concentrations of Filipinos may yield different results. Additionally, our convenience sampling yielded participants who were mostly employed and educated. Therefore we recommend using other sampling methods to further explore the relationship of the variables among foreign-born Filipino women who are unemployed, homemakers, those who are unable to work, and those with limited or no formal education. Our findings should be considered preliminary and hypothesis generating. While preliminary, however, our current findings provide foundational evidence about the relationship between resilience and acculturative stress among Filipino American women.

Another limitation was that the cross-sectional nature of this study does not allow for a causal understanding of the relationships between acculturative stress and resilience on FNAD. While our findings demonstrate significant relationships between resilience and acculturative stress, and between acculturative stress and family norms, causal directionality cannot be directly assessed in this cross-sectional study. Hence, our findings only demonstrate directionality of the variables in the sense of direct or inverse relationships, and in the context of our proposed theoretical model. Our findings are meant to be hypothesis generating rather than conclusive, as is expected when using an experimental or quasi-experimental design. Further longitudinal research may help parse the causal nature of these relationships.

Lastly, the study involved self-report surveys. Participants could have provided socially desirable answers, particularly in response to questions about acculturative stress and family norms. Social desirability may be related to underreporting mental health problems among Filipinos in order to prevent shame on their families and the loss of social acceptance (Gong et al., 2003). Adding measures such as a social desirability scale (Crowne and Marlowe, 1960), similar to what Gee et al. (2007) used in their research of Asian Americans, may be used in future research to address this issue.

## Conclusions

In spite of potential limitations, this study is the first of which we are aware to investigate the relationships between resilience, acculturative stress, and FNAD among Filipino American women. Previous studies involving resilience, acculturative stress, and the disclosure of mental health problems among Filipino American women are aggregated under the umbrella of Asian Americans. Aggregating different Asian ethnicities into one racial group overlooks the critical nuances of the heterogeneity of these subgroups (Museus and Truong, 2009). Filipino Americans have cultural values and historical backgrounds that are distinct from other Asian ethnicities; hence, they may also have different responses to stress associated with acculturation to U.S. Western culture. The findings of this study confirm the need to develop resilience interventions and resources that ameliorate acculturative stress and promote the disclosure and reporting of mental health problems among Filipino American women. Thus, our study offers new evidence on resilience and acculturative stress among Filipino American women, expands the literature and empirical evidence on the resilience of Asian American women and of Filipino American women in particular, and provides implications when developing resilience interventions for Filipino Americans.

## Declaration of Conflicting Interests

The authors declared no potential conflicts of interest concerning the research, authorship, or publication of this article.

Funding: This study was funded by the University of Nevada, Las Vegas (UNLV) School of Nursing Dean Research Support Fund.

## Funding

This study was funded by the University of Nevada, Las Vegas (UNLV) School of Nursing Dean Research Support Fund.
